# Supplementary material for: Inducible expression of interleukin-12 augments the efficacy of affinity-tuned chimeric antigen receptors in murine solid tumor models
Source: Nat Commun. 2023 Apr 12;14:2068. doi: 10.1038/s41467-023-37646-y (PMC10097865; doi:10.1038/s41467-023-37646-y)
Supplement: Supplementary file 1 — Supplementary Information [file 41467_2023_37646_MOESM1_ESM.pdf]

Inducible expression of interleukin-12 augments the efficacy of affinity-tuned chimeric antigen receptors in murine solid tumor models

Yanping Yang[1], Huan Yang[2], Yago Alcaina[1], Janusz Puc[2], Alyssa Birt[2], Yogindra Vedvyas[1], Michael Gallagher[2], Srinija Alla[2], Maria Cristina Riascos[1,3], Jaclyn E. McCloskey[1], Karrie Du[2], Juan Gonzalez-Valdivieso[1], Irene M. Min[3], Elisa de Stanchina[4], Matt Britz[2], Eric von Hofe[2], Moonsoo M. Jin[1,3]*

[1]Molecular Imaging Innovations Institute, Department of Radiology, Weill Cornell Medicine, New York, NY 10065, USA

[2]AffyImmune Therapeutics, Inc., Natick, MA 01760, USA

[3]Department of Surgery, Weill Cornell Medicine, New York, NY 10065, USA

[4]Antitumor Assessment Core Facility, Memorial Sloan Kettering Cancer Center, New York, NY 10065, USA

Address correspondence to: Moonsoo M. Jin, Department of Radiology, Weill Cornell Medical College, BB-1500, 413 E. 69th St., New York, NY 10065, USA. Phone: 646.962.6115; E-mail: moj2005@med.cornell.edu.

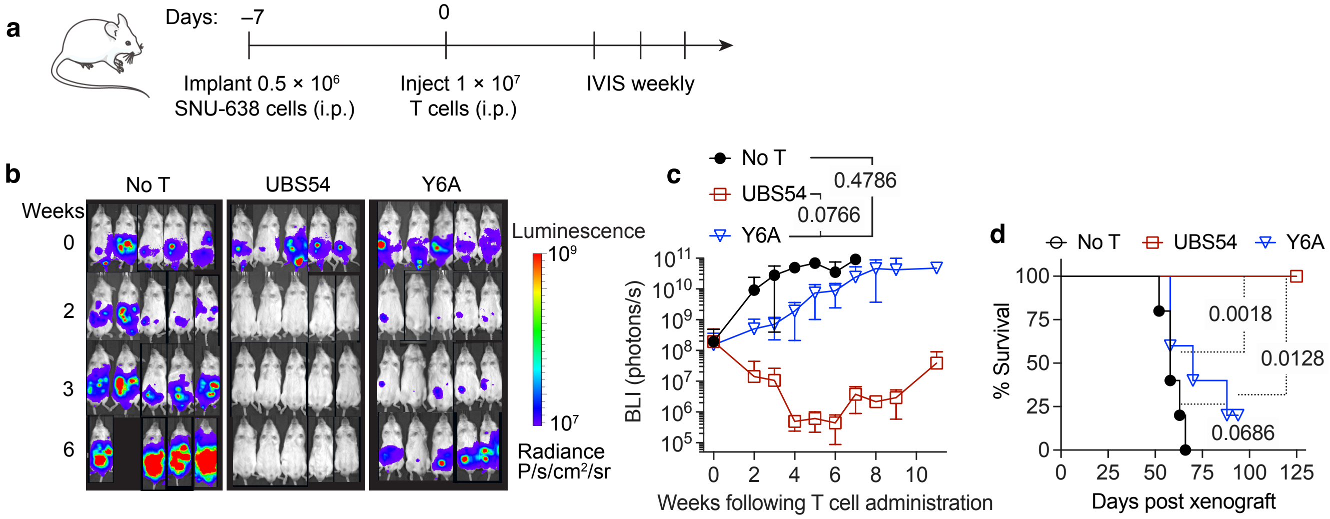

Supplementary Fig. 1 | Y6A CAR-T cells are weakly efficacious against intraperitoneal gastric tumors *in vivo*. a Schematic of the intraperitoneal SNU-638 tumor model. NSG mice were intraperitoneally injected with 0.5 × 10^6 SNU-638 cells and treated with UBS54 or Y6A CAR-T cells (1 × 10^7 cells/mouse, i.p.) or left untreated (No T) 7 days after implantation. **b** Bioluminescence images of mice at indicated time points after CAR-T cell treatment. **c** Tumor burden quantified by total body bioluminescence intensity. Data are shown as mean ± SD (*n* = 5 biological independent mice per group in one experiment). Statistical significance was determined by two-way ANOVA with Tukey's multiple comparisons test. **d** Kaplan–Meier survival curves (*n* = 5 biological independent mice per group). Log-rank (Mantel–Cox) test.

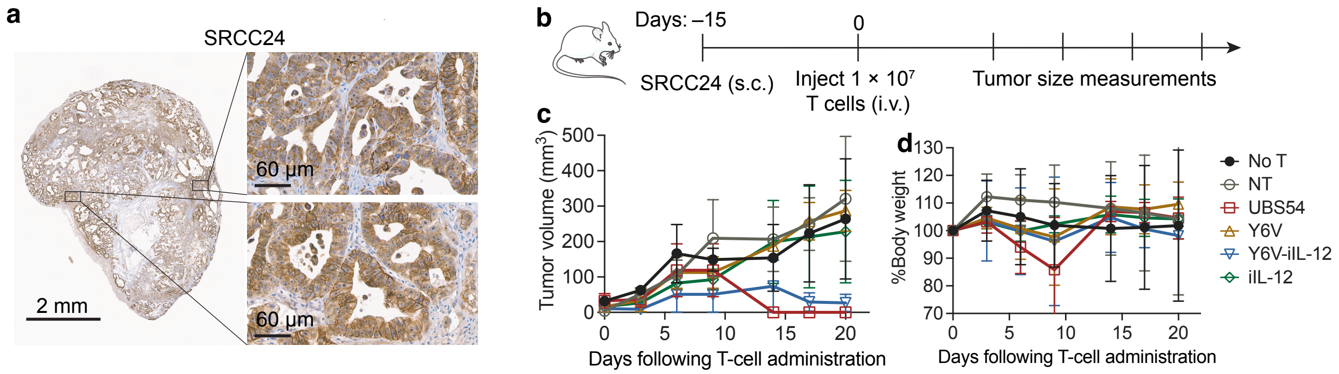

Supplementary Fig. 2 | Y6V-iIL12 CAR-T cells induced regression of patient-derived xenografts of GC. a Expression of EpCAM on SRCC24 PDX tumor. **b** Schematic of the experimental design using SRCC24 PDX model. **c** Volumes of SRCC24 tumor in NSG mice without treatment (No T) or treated with CAR-T cells (No T, *n* = 2 mice; non-transduced T (NT) and Y6V-iIL-12, *n* = 3 mice; UBS54, *n* = 5 mice; Y6V and iIL-12, *n* = 4 mice in one experiment). **d** Body weight changes relative to baseline. Data represent mean ± SD. Allogenic CAR-T cells from healthy donors were used in this study.

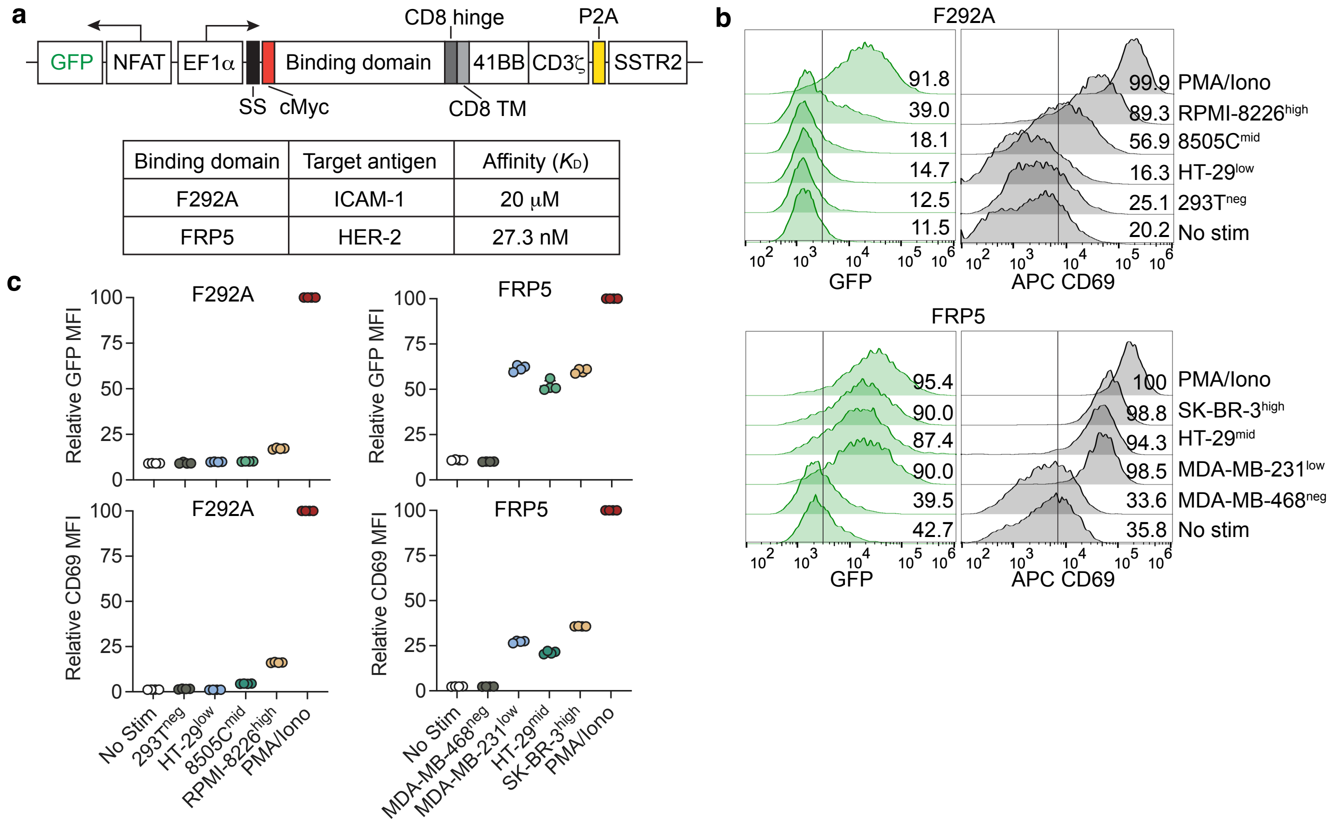

Binding domain	Target antigen	Affinity (K_D)
F292A	ICAM-1	20 μM
FRP5	HER-2	27.3 nM

Supplementary Fig. 3 | NFAT activation in ICAM-1-specfic F292A CAR-T and HER-2-sepcfic FRP5 CAR-T cells. a Lentiviral vectors encoding F292A-iGFP and FRP5-iGFP CARs. The binding affinity of FRP5 CAR was determined by a saturation binding assay using AF647-conjugated monomeric HER-2. The affinity of F292A was determined previously by a flow cytometry-based competition binding assay. **b** Flow cytometric quantification of CAR-mediated NFAT activation, as indicated by NFAT-driven GFP expression in Jurkat CAR-T cells following stimulation for 24 h with target cells. CD69 was used as a marker for T-cell activation. Numbers indicate the percentages of live, single cells in the GFP- or CD69-positive gate. Neg, low, mid and high represent negative-, low-, medium-, and high-EpCAM expression, respectively. **c** Relative mean fluorescence intensity (MFI) of GFP and CD69 expression from **b**. Data were normalized to PMA/Ionomycin treatment and were presented as mean ± SD of quadruplicate samples.

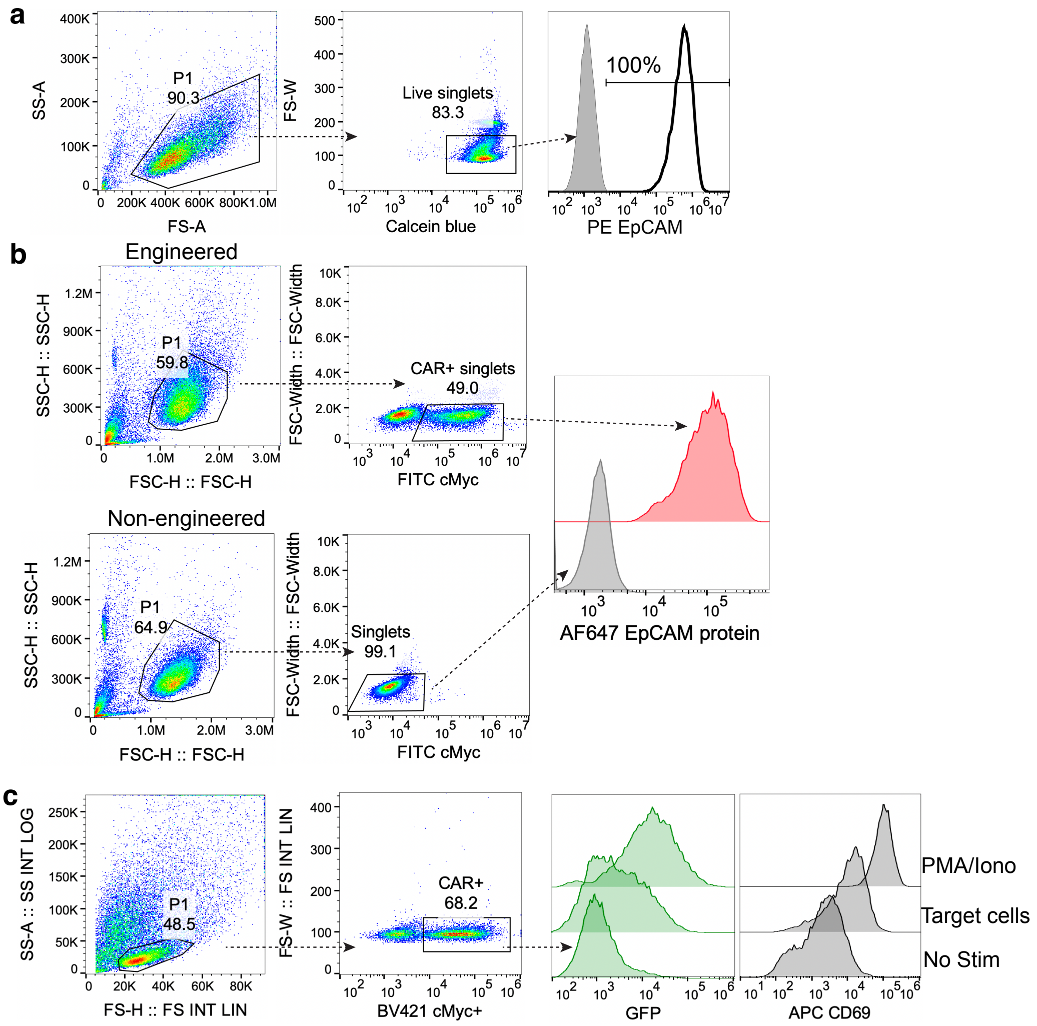

Supplementary Fig. 4 | The general gating strategy for flow cytometry. a Gating strategy for antigen expression in tumor cell lines and primary EpC (data shown in Fig. 1c). Cells were gated based on size and granularity (FS-A × SS-A). Live singlets were gated by FS-W and calcein blue and evaluated for expression of EpCAM. **b** Gating strategy for relative EpCAM binding (data shown in Fig. 1b). Cells were gated by FS-H × SS-H, and then gated for CAR+ live singlets by FS-W and cMyc staining. CAR+ cells were evaluated for binding to EpCAM protein. Non-engineered cells were used as negative controls. **c** Gating strategy for NFAT-GFP activation and T-cell activation (Data shown in Fig. 5b and Supplementary Fig. 3b). Cells were gated by FS-H × SS-A, and then gated for CAR+ live singlets by FS-W and cMyc staining. CAR+ cells were evaluated for GFP and CD69 expression.
