# Supplementary material for: Inducible expression of interleukin-12 augments the efficacy of affinity-tuned chimeric antigen receptors in murine solid tumor models
Source: Nat Commun. 2023 Apr 12;14:2068. doi: 10.1038/s41467-023-37646-y (PMC10097865; doi:10.1038/s41467-023-37646-y)
Supplement: Supplementary file 3 — Reporting Summary [file 41467_2023_37646_MOESM3_ESM.pdf]

nature portfolio

Corresponding author(s): Moonsoo M. Jin

Last updated by author(s): Mar 12, 2023

Reporting Summary

Nature Portfolio wishes to improve the reproducibility of the work that we publish. This form provides structure for consistency and transparency in reporting. For further information on Nature Portfolio policies, see our Editorial Policies and the Editorial Policy Checklist.

Statistics

For all statistical analyses, confirm that the following items are present in the figure legend, table legend, main text, or Methods section.

n/a	Confirmed	
☐	☒	The exact sample size (*n*) for each experimental group/condition, given as a discrete number and unit of measurement
☐	☒	A statement on whether measurements were taken from distinct samples or whether the same sample was measured repeatedly
☐	☒	The statistical test(s) used AND whether they are one- or two-sided<br>*Only common tests should be described solely by name; describe more complex techniques in the Methods section.*
☒	☐	A description of all covariates tested
☐	☒	A description of any assumptions or corrections, such as tests of normality and adjustment for multiple comparisons
☐	☒	A full description of the statistical parameters including central tendency (e.g. means) or other basic estimates (e.g. regression coefficient) AND variation (e.g. standard deviation) or associated estimates of uncertainty (e.g. confidence intervals)
☐	☒	For null hypothesis testing, the test statistic (e.g. *F*, *t*, *r*) with confidence intervals, effect sizes, degrees of freedom and *P* value noted<br>*Give P values as exact values whenever suitable.*
☒	☐	For Bayesian analysis, information on the choice of priors and Markov chain Monte Carlo settings
☒	☐	For hierarchical and complex designs, identification of the appropriate level for tests and full reporting of outcomes
☒	☐	Estimates of effect sizes (e.g. Cohen's *d*, Pearson's *r*), indicating how they were calculated

Our web collection on statistics for biologists contains articles on many of the points above.

Software and code

Policy information about availability of computer code

Data collection	Gallios Cytometry List Mode Data Acquisition & Analysis Software v.1.2<br>RTCA software Pro v.2.6.1<br>Living Image v.4.7.2<br>Siemens Inveon micro-PET/CT Acquisition Workplace v.1.5
Data analysis	FlowJo v.10.8.1<br>RTCA software Pro v.2.6.1<br>Living Image v.4.7.2<br>AMIDE v.1.0.5<br>GraphPad Prism 9 v.9.5.1<br>Aperio ImageScope v.12.4.3

For manuscripts utilizing custom algorithms or software that are central to the research but not yet described in published literature, software must be made available to editors and reviewers. We strongly encourage code deposition in a community repository (e.g. GitHub). See the Nature Portfolio guidelines for submitting code & software for further information.

Data

Policy information about availability of data

All manuscripts must include a data availability statement. This statement should provide the following information, where applicable:

- Accession codes, unique identifiers, or web links for publicly available datasets
- A description of any restrictions on data availability
- For clinical datasets or third party data, please ensure that the statement adheres to our policy

The main data supporting the results of this study are available within the paper and its supplementary information files. Source data are provided with this paper.

Human research participants

Policy information about studies involving human research participants and Sex and Gender in Research.

Reporting on sex and gender	N/A
Population characteristics	N/A
Recruitment	N/A
Ethics oversight	N/A

Note that full information on the approval of the study protocol must also be provided in the manuscript.

Field-specific reporting

Please select the one below that is the best fit for your research. If you are not sure, read the appropriate sections before making your selection.

☒ Life sciences ☐ Behavioural & social sciences ☐ Ecological, evolutionary & environmental sciences

For a reference copy of the document with all sections, see nature.com/documents/nr-reporting-summary-flat.pdf

Life sciences study design

All studies must disclose on these points even when the disclosure is negative.

Sample size	Sample sizes were not predetermined. In vitro experiments were performed in triplicate or otherwise noted. For in vivo animal experiments, sample sizes were based on previous experience (PMID: 34341066) and pilot studies, with an effort to achieve a minimum of n = 4, mostly n ≥ 5 mice per cohort, which proved sufficient to determine the reproducibility.
Data exclusions	No data were excluded from data analyses.
Replication	Attempts at replication were highly successful. Most of the animal studies were performed independently at least twice, with T cells from different donors, and the data were pooled. Detailed information are provided in the relevant figure legends.
Randomization	All in vitro samples and controls were treated side-by-side using the identical protocols, so no randomization was used. For in vivo experiments, mice were randomly assigned to different treatment groups after confirmation of successful tumor engraftment by bioluminescence imaging or tumor size measurements.
Blinding	The investigators were not blinded for in vitro studies due to the feasibility during data collection. Animal studies were performed by technicians who were not blinded to group allocation to avoid any mislabeling of different treatment groups. Compared samples were collected and analyzed under the same conditions. All measurements were made objectively.

Reporting for specific materials, systems and methods

We require information from authors about some types of materials, experimental systems and methods used in many studies. Here, indicate whether each material, system or method listed is relevant to your study. If you are not sure if a list item applies to your research, read the appropriate section before selecting a response.

Materials & experimental systems

n/a	Involved in the study
☐	☒ Antibodies
☐	☒ Eukaryotic cell lines
☒	☐ Palaeontology and archaeology
☐	☒ Animals and other organisms
☒	☐ Clinical data
☒	☐ Dual use research of concern

Methods

n/a	Involved in the study
☒	☐ ChIP-seq
☐	☒ Flow cytometry
☒	☐ MRI-based neuroimaging

Antibodies

Antibodies used	Anti-human EpCAM antibody (Agilent, clone Ber-EP4, cat. no. M0804) was used for IHC staining.<br><br>The following antibodies were used to stain cells for flow cytometry analysis:<br>PE human CD326 (EpCAM) antibody (BioLegend, clone 9C4, cat. no. 324206, 1:100 dilution)<br>APC human CD54 (ICAM-1) antibody (BioLegend, clone HA58, cat. no. 353112, 1:100 dilution)<br>APC human CD340 (HER-2) antibody (BioLegend, clone 24D2, cat. no. 324408, 1:100 dilution)<br>FITC c-myc antibody (Miltenyi Biotec, clone SH1-26E7.1.3, cat. no. 130-116-485, 1:50 dilution)<br>Pacific Blue human CD3 antibody (BioLegend, clone HIT3a, cat.no.300330, 1:50 dilution)<br>APC human CD69 antibody (BioLegend, clone FN50, cat.no. 310910, 1:50 dilution)<br>Mouse IgG (Sigma-Aldrich, cat. no. I8765, 200 µg/mL)
Validation	All used antibodies were validated with appropriate positive and negative controls such as non-transduced T cells. Antibody validation by the manufacturer is available at each manufacturer's website.

Eukaryotic cell lines

Policy information about cell lines and Sex and Gender in Research

Cell line source(s)	The human breast cancer cell lines MDA-MB-231, MDA-MB-468, and SK-BR-3, human colon cancer cell line HT-29, human pancreatic cancer cell line SW-1990, human myeloma cell line RPMI-8226, and cell lines 293T and Jurkat were purchased from the American Type Culture Collection (ATCC). Human gastric cancer cell line MKN-45 was purchased from DSMZ, whereas cell line SNU-638 was obtained from the Korean Cell Line Bank (Seoul National University, Seoul, Korea). Human thyroid cancer cell line 8505C was purchased from Sigma.
Authentication	Certificate of Analysis was provided with cell lines by the vendors, and authentication was conducted by ATCC DNA fingerprinting. Morphology and antigen expressions were confirmed routinely by flow cytometry.
Mycoplasma contamination	The cell lines were tested by the vendor and also tested routinely using a MycoAlert detection kit (Lonza). The cell lines were tested to be negative for mycoplasma prior to use.
Commonly misidentified lines<br>(See ICLAC register)	No commonly misidentified cell lines were used.

Animals and other research organisms

Policy information about studies involving animals; ARRIVE guidelines recommended for reporting animal research, and Sex and Gender in Research

Laboratory animals	Four to six weeks old male NOD.Cg-Prkdcscid Il2rgtm1Wjl/SzJ (NSG) mice were purchased from The Jackson Laboratory (Stock # 005557). All experimental mice were co-housed in the Animal Core Facility at Weill Cornell Medicine (New York, NY) under specific pathogen-free conditions and provided with sterile food and water. They were subjected to a 12 h light/dark cycle and maintained at an ambient temperature of 21–27°C and a humidity of 40–60%.
Wild animals	This study did not involve wild animals.
Reporting on sex	Sex was not considered in study design.
Field-collected samples	This study did not involve samples collected from the field.
Ethics oversight	All procedures involving animals were approved by the Institutional Animal Care and Use Committee at Weill Cornell Medicine.

Note that full information on the approval of the study protocol must also be provided in the manuscript.

Flow Cytometry

Plots

Confirm that:

- [x] The axis labels state the marker and fluorochrome used (e.g. CD4-FITC).
- [x] The axis scales are clearly visible. Include numbers along axes only for bottom left plot of group (a 'group' is an analysis of identical markers).
- [x] All plots are contour plots with outliers or pseudocolor plots.
- [x] A numerical value for number of cells or percentage (with statistics) is provided.

Methodology

Sample preparation	Cells were washed with PBS containing 0.5% BSA and blocked with 200 µg/ml mouse IgG (Sigma-Aldrich, cat. no. I8765) prior to staining. Cell staining was performed at 4°C in the dark for 15 minutes. Cells were washed twice and stained with Calcein Blue (Sigma-Aldrich, cat. no. M1255) before analysis.
Instrument	Gallios Flow Cytometer (BECKMAN COULTER)
Software	Flow cytometry data were collected using Gallios Cytometry List Mode Data Acquisition & Analysis Software v.1.2 and were analyzed using FlowJo v.10.8.1
Cell population abundance	We did not perform any flow cytometric sorting for tumor cell lines or T cells. During CAR-T cell manufacturing, T cells were enriched by immunomagnetic sorting using a combination of anti-CD4- and anti-CD8-conjugated microbeads (Miltenyi Biotec). No sorting was performed after CAR-T cell manufacturing. The purity of CD3+ T cells and the abundance of CAR+ T cells were analyzed by flow cytometry.
Gating strategy	The desired cell population was selected using forward and side scatter. Live single cells were selected by Calcein Blue staining and using forward scatter width linearity. Boundaries between positive and negative staining cell populations were based on unstained controls or non-transduced cell controls.

- [x] Tick this box to confirm that a figure exemplifying the gating strategy is provided in the Supplementary Information.
